# Antimycobacterial Activity of Laurinterol and Aplysin from *Laurencia johnstonii*

**DOI:** 10.3390/md18060287

**Published:** 2020-05-30

**Authors:** Sara García-Davis, Karla Leal-López, Carmen A. Molina-Torres, Lucio Vera-Cabrera, Ana R. Díaz-Marrero, José J. Fernández, Pilar Carranza-Rosales, Ezequiel Viveros-Valdez

**Affiliations:** 1 Instituto Universitario de Bio-Orgánica Antonio González (IUBO AG), Departamento de Química Orgánica, Universidad de La Laguna (ULL), Avda. Astrofísico F. Sánchez, 2, 38206 La Laguna, Tenerife, Spain; sgdavis@ull.es (S.G.-D.); adiazmar@ull.edu.es (A.R.D.-M.); jjfercas@ull.edu.es (J.J.F.); 2 Facultad de Ciencias Biológicas (FCB), Universidad Autónoma de Nuevo León (UANL), Av. Pedro de Alba s/n, 66450 San Nicolás de los Garza, Nuevo León, Mexico; karla.leallp@uanl.edu.mx; 3 Servicio de Dermatología, Hospital Universitario “José E. González”, Universidad Autónoma de Nuevo León (UANL), Madero y Gonzalitos, Col. Mitras Centro, 64460 Monterrey, Nuevo León., Mexico; carmen.molinatr@uanl.edu.mx (C.A.M.-T.); lucio.veracb@uanl.edu.mx (L.V.-C.); 4 Centro de Investigación Biomédica del Noreste (CIBIN), Instituto Mexicano del Seguro Social (IMSS), Calle Jesús Dionisio González # 501, Col. Independencia, 64720 Monterrey, Nuevo León, Mexico; carranza60@yahoo.com.mx

**Keywords:** marine natural products, *Laurencia*, brominated sesquiterpenes, antimycobacterial, nontuberculous mycobacteria, tuberculosis

## Abstract

Marine environments represent a great opportunity for the discovery of compounds with a wide spectrum of bioactive properties. Due to their large variety and functions derived from natural selection, marine natural products may allow the identification of novel drugs based not only on newly discovered bioactive metabolites but also on already known compounds not yet thoroughly investigated. Since drug resistance has caused an increase in infections by *Mycobacterium tuberculosis* and nontuberculous mycobacteria, the re-evaluation of known bioactive metabolites has been suggested as a good approach to addressing this problem. In this sense, this study presents an evaluation of the in vitro effect of laurinterol and aplysin, two brominated sesquiterpenes isolated from *Laurencia johnstonii*, against nine *M. tuberculosis* strains and six nontuberculous mycobacteria (NTM). Laurinterol exhibited good antimycobacterial activity, especially against nontuberculous mycobacteria, being remarkable its effect against *Mycobacterium abscessus*, with minimum inhibitory concentration (MIC) values lower than those of the reference drug imipenem. This study provides further evidence for the antimycobacterial activity of some sesquiterpenes from *L. johnstonii*, which can be considered interesting lead compounds for the discovery of novel molecules to treat NTM infections.

## 1. Introduction

Antimicrobial resistance is a global health problem that increases the difficulties in treating common infectious diseases and causes a significant increment in healthcare costs, with concomitant lengthier stays in hospitals and intensive care requirements [1].

Tuberculosis (TB), caused by *Mycobacterium tuberculosis* (Mtb), is one of the top 10 causes of death worldwide. In 2018, 1.5 million people died from this disease, with over 95% of cases occurring in developing countries [2].

TB can be treated effectively by using the first-line drugs (FLDs) isoniazid, rifampin, pyrazinamide, ethambutol, and streptomycin. Nevertheless, multidrug-resistant Mtb strains (MDR-TB) pose a growing problem that requires the use of second-line drugs, that are not easy to procure and are more toxic and expensive than FLDs [3]. WHO estimates that, in 2018, there were about 484,000 new cases of TB with resistance to rifampicin, of which 78% were caused by MDR-TB, and only 56% are currently successfully treatable [2]. In addition to Mtb, there are over 170 different species of mycobacterial pathogens defined as nontuberculous mycobacteria (NTM) that cause a wide spectrum of diseases that include TB-like pulmonary, extra-pulmonary, and disseminated diseases. Often, NTM do not respond to TB treatments, and usually, lengthy and complex drug therapies are required to treat them. Furthermore, most NTM have become resistant to many antimicrobials [4]. 

Natural products are considered privileged molecules because they are fashioned by natural selection to interact with cellular targets with high efficiency and selectivity and to avoid resistance. Due to these characteristics, natural products and their derivatives represent over one-third of all FDA-approved new molecular entities; in particular, 69% of all antibacterial agents originated from natural products [5]. Nonetheless, the total amount of approved compounds barely represents over 0.1% of the more than 25,000 known antibiotic natural products, mainly due to issues concerning their efficacy, toxicity, or stability, among others. Given the efficacy and safety indexes exhibited by the first successfully employed natural drugs, criteria governing novel antibiotic discovery are strict, making it difficult to identify more successful candidates. Due to the pressing need to identify new antibiotic compounds, re-evaluation of known molecules has been suggested [6].

Marine organisms have become an important source of several thousands of bioactive compounds [7]. Eight of these compounds have gained clinical approval, and around 30 are studied in different clinical phases of drug development. Nevertheless, none of them have been exploited as antibacterial drugs [8]; however, some approved compounds are under study for the treatment of severe pain, hypertriglyceridemia, schizophrenia, cognitive disorders, and viral infections [8,9].

**Figure 1:**
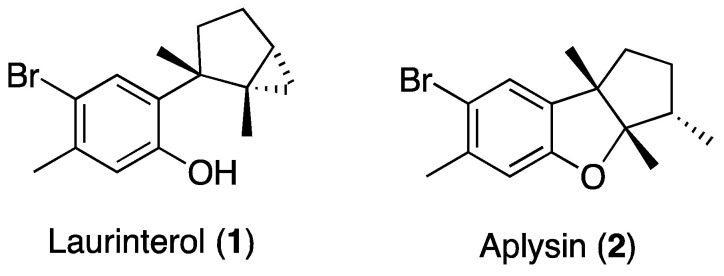
Structures of the natural sesquiterpenes laurinterol and aplysin.

Among marine organisms, algae of the genus *Laurencia* are considered one of the richest sources of novel compounds with a wide range of bioactive properties, such as the brominated sesquiterpenes laurinterol (**1**) and aplysin (**2**) (Figure 1) [10]. Laurinterol has been evaluated for its antibacterial [11,12,13], cytotoxic [13,14,15], antifouling [16], anti-*Acanthamoeba* [17], Na/K ATPase inhibition [18], insecticidal, and repellent properties [19]. Meanwhile, aplysin has shown moderate cytotoxic activity and has been evaluated for its mechanisms of action in different biological models [20,21,22,23].

Recently, membrane proteins, such as P-type ATPases and ATP synthases, have been suggested as promising targets to treat mycobacterial infections, making it possible to overcome the limitations caused by the lipid-rich cell of mycobacteria [24,25]. In this sense, laurinterol has been reported as a potent Na/K ATPase inhibitor [18], which encouraged us to explore its antimycobacterial properties as well as to evaluate those of aplysin, a related structural compound, abundant and with low toxicity, and to explore the possibility of obtaining it from laurinterol [17]. 

## 2. Results and Discussion

The emergence of drug resistance is one of the challenges to control TB and other mycobacterial infections, resulting in the evident need to develop new drugs. Several new drugs and compounds are currently undergoing clinical evaluation as treatment options against mycobacterial infections [26,27,28,29]. Taking into consideration the important role of natural products and their semi-synthetic derivatives as drug leads, it is not surprising that some antimicrobials such as rifampicin, streptomycin, amikacin, viomycin, kanamycin, capreomycins, and cycloserine are currently used in combination with other antitubercular agents in the treatment of mycobacterial infections [30]. 

More than 170 compounds isolated from marine organisms, mostly from microorganisms and invertebrates, have been reported to possess anti-TB properties. They include several biosynthetic classes such as alkaloids, peptides, terpenoids, sterols, and others. Despite that, only 10 of those compounds are considered candidates for further development, presenting minimum inhibitory concentration (MIC) values below 64 μg/mL and a selectivity index (SI, CC_50_/MIC) higher than 10 [31]. 

Table 1 shows the activity of laurinterol and aplysin against nine *M. tuberculosis* strains, in comparison to that of rifampicin, the most common drug for TB treatment [32], used as positive control. Laurinterol inhibited eight out of the nine strains of Mtb, with MICs ≤ 100 µg/mL. *M. tuberculosis* CIPTIR-F296 was the most susceptible strain for both compounds; laurinterol was more active, with **a** MIC value of 25 µg/mL.

In comparison with TB treatments, anti-NTM therapies have been remarkably neglected. Hence, it has been suggested to screen known anti-Mtb compounds against strains of NTM [33]. For this reason, we evaluated laurinterol and aplysin against six clinical isolates of NTM (Table 2): three strains of *Mycobacterium abscessus*, one strain of *Mycobacterium fortuitum*, and two strains of *Mycobacterium intracellulare*. Both compounds were active against NTM; whereas aplysin inhibited *M. intracellulare* LIID-01 with MIC of 50 µg/mL, laurinterol exhibited good activity against all the strains tested, particularly against *M. abscessus* strains (MIC 6.2 µg/mL). It is important to emphasize that *Mycobacterium avium* complex (*M. avium* and *M. intracellulare*) and *M. abscessus* represent prevalent sources of infections and, in addition to the lengthy and complex treatment they require (18–24 months with at least three drugs in combination), they are resistant to many common antibiotics [28]. In this study, imipenem and linezolid were selected as pharmacological controls because they are part of the recommended treatment for NTM infections [34,35,36,37].

Previously, laurinterol and aplysin were evaluated for their toxicity against murine macrophages and they exhibited half-maximal cytotoxic concentration (CC_50_) of 23.7 and 323.7 μg/mL, respectively [17]. In our study, given the antimycobacterial activity of sesquiterpenes, laurinterol exhibited an SI of 3.8 for *M. abscessus*, whereas aplysin showed an SI between 3.2 and 6.5 for two strains of *M. intracellulare* and *M. abscessus* and one strain of *M. tuberculosis*. The SI is used to estimate the therapeutic window of a drug and to identify drug candidates for further studies. An SI value less than 2 indicates general toxicity of the pure compound, therefore both laurinterol and aplysin were considered good drug candidates and subjected to subsequent analysis.

**Figure 2:**
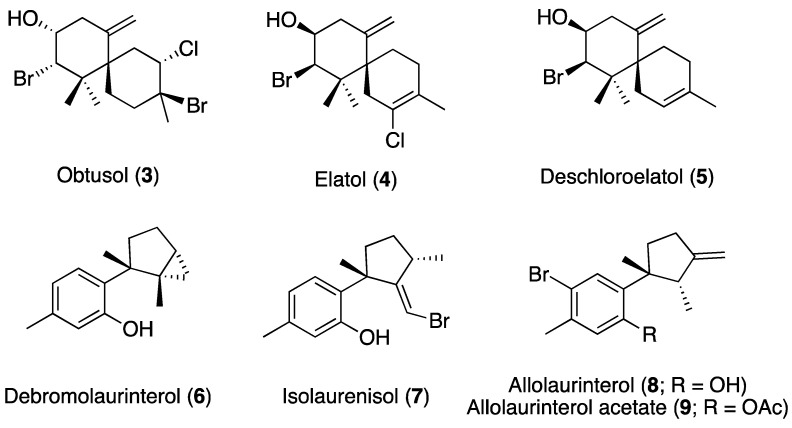
*Laurencia*-derived sesquiterpenes evaluated for their antimycobacterial activity.

To date, algae metabolites represent over 2% of the marine anti-TB compounds reported. Among the metabolites isolated from *Laurencia* that have been tested against Mtb (Figure 2), obtusol (**3**), elatol (**4**), and deschloroelatol (**5**) exhibited MICs of 32 µg/mL, debromolaurinterol (**6**) and isolaurenisol (**7**) showed MICs of 64 and 38 µg/mL, respectively, and allolaurinterol (**8**) and allolaurinterol acetate (**9**) presented MICs of 16 and 9 µg/mL, respectively [38,39,40]. Axisonitrile-3, a sesquiterpene isonitrile isolated from the sponge *Acanthella klethra*, is one of the most active marine compounds against Mtb and exhibited a MIC of 2 µg/mL [38].

Despite the urge to find new anti-NTM treatments, marine natural products have been weakly evaluated; Biá Ventura et al. investigated the antimycobacterial activity of two extracts of *Laurencia dendroidea* and three of its halogenated sesquiterpenes against *Mycobacterium bovis*, noting obtusol (**3**) with a half-maximal inhibitory concentration (IC_50_) of 31.4 µg/mL [39].

*Laurencia*-derived sesquiterpenes with chamigrane (compounds **3**–**5**), cyclolaurane (compound **6**), and laurane (compounds **7**–**9**) skeletons have shown antitubercular properties. The presence of lipophilic groups such as an exocyclic double bond, an aromatic ring, or halogenated substitutions—particularly bromine atoms—as well as the presence of a hydroxyl group are common structural features that all these small active molecules share. These structural characteristics are also found in laurinterol and aplysin, which present cyclolaurane and laurane frameworks, respectively. However, despite the bromide atom, the formation of an ether bond between the aromatic ring and the cyclopentane fragment of aplysin decreases its antimycobacterial activity, revealing the relevance of the hydroxyl group for this activity.

In this work, we observed that the brominated cyclolaurane laurinterol can be considered an interesting lead compound for the discovery of novel antimycobacterial molecules to treat NTM infections. One of the major challenges in the biodiscovery process is the availability of bioactive compounds; however, this problem does not occur for laurinterol since it is the major metabolite found in *Laurencia johnstonii*, with an estimated abundance of 70% in the total crude extract. These results are of remarkable importance, since NTM infections, especially those due to *M. avium* and *M. abscessus* complexes, have increased worldwide. This is an important concern for healthcare due to the intrinsic resistance of these NTM to most conventionally utilized antimicrobials and to *M. abscessus* being one of the most drug-resistant mycobacteria [41]. Additionally, person-to-person transmission of *M. abscessus* among cystic fibrosis patients has been reported [42], overturning the general belief that NTM infections are acquired through environmental sources. This poses a major challenge for drug development, where hit rates in primary screens for *M. abscessus* can be lower than 0.1%. Thus, the generation of attractive lead compounds represents a bottleneck [43].

In the search for potential compounds with anti-TB properties, those identified as active have a MIC of 50–64 µg/mL. Nonetheless, less active or inactive derivatives are also recommended to be revisited if they highlight structure–activity relationships, in order to increase the opportunities to identify new antimycobacterial treatments [44,45]. Sesquiterpenes from *L. johnstonii* can be considered interesting lead compounds as model scaffolds for the development and discovery of more efficient antimycobacterial molecules to treat NTM infections, reinforcing the fact that anti-NTM compounds do not have to be necessarily active against Mtb strains. 

The reinvestigation of known natural compounds and their synthetic derivatives will open opportunities to reveal yet undiscovered scaffolds as drug leads. In this context, the vast chemical diversity of marine natural products represents an immense opportunity to be exploited, since a systematic study of chemical compounds able to provide novel scaffolds for further optimization is greatly needed.

## 3. Materials and Methods 

### 3.1. Extraction and Isolation 

Ethanolic extract of *L. johnstonii* collected in Baja California Sur, México, was chromatographed on Sephadex LH-20 in order to obtain five fractions. Fraction 3 was rechromatographed using Flash Silicagel and a stepwise gradient from *n*-hexane to ethyl acetate. Fraction 3.2 (95% *n*-hexane) was partitioned on a Silicagel open column using a stepwise gradient from *n*-hexane to ethyl acetate to yield pure laurinterol and aplysin, as previously described [17]. 

### 3.2. Mycobacterial Strains and Culture Conditions

Six clinical isolates of NTM, including three strains of *M. abscessus*, two of *M. intracellulare*, and one of *M. fortuitum*, as well as nine strains of *M. tuberculosis* were obtained from the Laboratorio Interdisciplinario y de Investigación Dermatológica (LIID) belonging to the Hospital Universitario José E. González (Monterrey, Nuevo León, México). All the strains were identified to the species level by a biochemical test or by PCR restriction analysis of a 441-bp sequence (Telenti fragment) of the *hsp65* gene, as previously described [46]. The susceptibility to first-line drugs of all *M. tuberculosis* strains was determined by the proportion method, being five of them multidrug-resistant. The reference isolate *M. tuberculosis* H37Rv ATCC 27294 (GeneBank accession number NC_000962, downloaded from NCBI) was obtained from the American Type Culture Collection (ATCC); strain CDC1551 was donated by Dr. F. Quinn, University of Georgia, Anthens, GA (GeneBank accession number AE000516.2, downloaded from NCBI). The strains were activated from the frozen stocks in Lowenstein–Jensen Medium and Blood Agar by incubation at 37 °C for 7–14 days.

### 3.3. Determination of the Minimum Inhibitory Concentration (MIC)

MIC, defined as the minimal concentration of compound which prevents the visible growth of bacteria, was determined by measurement of turbidity in wells using the broth microdilution method [47]. The susceptibility of Mtb to laurinterol and aplysin was evaluated using Alamar Blue [48]; *M. tuberculosis* H37Rv was used as a susceptible-strain control. The antibiotic rifampicin was also tested for activity comparison.

Cation-adjusted Mueller–Hinton broth (CA-MHB) was used for rapidly growing mycobacteria (*M. abscessus* and *M. fortuitum*), and Middlebrook 7H9 broth supplemented with oleic albumin dextrose catalase (OADC) was used for slowly growing mycobacteria (*M. intracellulare*) [47]. MIC values were determinated after 72 h at 37 °C for *M. abscessus* and *M. fortuitum* strains, and after 7 to 10 days for *M. intracellulare* strains. *Staphylococcus aureus* ATCC 29213 was used as an external control, and the antibiotics linezolid and imipenem at concentrations established by the CLSI (maximum 64 μg/mL) were used for activity comparison. Both compounds were evaluated from 3.12 to 100 μg/mL. The experiments were performed in duplicate.

## 4. Conclusions

With the emerging threat of drug resistance, particularly for infections caused by NTM, our results highlight the relevance to revisit marine-sourced compounds as potential lead molecules for the development of new therapies. Laurinterol showed a moderate activity (MIC 25–50 µg/mL) against six out of the nine *M. tuberculosis* strains tested. However, it showed good activity (MIC 6.25–25 µg/mL) against all NTM strains tested. In the case of *M. abscessus* LIID-01, LIID-02, and LIID-03, as well as *M. intracellulare* LIID*-*02 and M*. tuberculosis* CIPTIR -F296, the MIC values either improved or were in the same range of those of the reference drug used as a positive control. Considering the abundance of laurinterol in the whole extract of *L. johnstonii* and its anti-mycobacterial activity, this study opens the path to research on laurinterol as a potential lead compound for the discovery of anti-NTM drugs and encourages further structure–activity relationship studies to evaluate its mode of action.

## Figures and Tables

**Table 1:** Minimum inhibitory concentration (MIC) values of laurinterol and aplysin for *Mycobacterium tuberculosis* strains.

Strain	Laurinterol µg/mL	Aplysin µg/mL	* Rifampicin µg/mL
*M. tuberculosis* H37Rv ATCC 27294	100	Nt	0.1
*M. tuberculosis* CDC 1551	50	Nt	0.1
*M. tuberculosis* LIID-28-99	50	Nt	0.1
*M. tuberculosis* LIID-582-15	25	>100	0.1
*M. tuberculosis* LIID-619-15	25	>100	1
*M. tuberculosis* LIID-853-15	100	>100	1
*M. tuberculosis* CIPTIR-F296	25	50	32
*M. tuberculosis* CIPTIR *-*D152	50	>100	1
*M. tuberculosis* CIPTIR -C131	>100	>100	0.1

Nt: Not tested; * reference drug.

**Table 2:** MIC values of laurinterol and aplysin for nontuberculous mycobacteria clinical strains.

Strain	Laurinterol µg/mL	Aplysin µg/mL	* Linezolid µg/mL	* Imipenem µg/mL
*Mycobacterium abscessus* LIID- 01	6.2	100	1	32
*M. abscessus* LIID- 02	6.2	100	1	32
*M. abscessus* LIID-03	6.2	>100	1	8
*Mycobacterium fortuitum* LIID- 01	12.5	>100	8	4
*Mycobacterium intracellulare* LIID-01	12.5	50	2	1
*M. intracellulare* LIID-02	25.0	100	8	>64

* Reference drugs.
